# A New Fuzzy Logic Classifier Based on Multiscale Permutation Entropy and Its Application in Bearing Fault Diagnosis

**DOI:** 10.3390/e22010027

**Published:** 2019-12-24

**Authors:** Wenhua Du, Xiaoming Guo, Zhijian Wang, Junyuan Wang, Mingrang Yu, Chuanjiang Li, Guanjun Wang, Longjuan Wang, Huaichao Guo, Jinjie Zhou, Yanjun Shao, Huiling Xue, Xingyan Yao

**Affiliations:** 1 College of Mechanical Engineering, North University of China, Taiyuan 030051, China; dwh@nuc.edu.cn (W.D.); s1802011@st.nuc.edu.cn (X.G.); wjy@nuc.edu.cn (J.W.); zhoujinjiechina@126.com (J.Z.); syj@nuc.edu.cn (Y.S.); s1802036@st.nuc.edu.cn (H.X.); 2 School of Mechanical and Electrical Engineering, North University of China, Taiyuan 030051, China; yumingrang@163.com; 3 School of Mechanical Engineering, Guizhou University, Guiyang 550025, China; chuanjiang_li@163.com; 4 State Key Laboratory of Marine Resource Utilization in South China Sea, Hainan University, Haikou 570228, China; juanywong@126.com; 5 Collage of Information and Communication Engineering, Hainan University, Haikou 570228, China; 6 School of Energy and Power Engineering, North University of China, Taiyuan 030051, China; ghc0716@163.com; 7 School of Computer Science and Information Engineering, Chongqing Technology and Business University, Chongqing 400067, China; xyyao@ctbu.edu.cn

**Keywords:** fault diagnosis, HMDSOF, harmonic mean difference, MPE, LDA

## Abstract

The self-organizing fuzzy (SOF) logic classifier is an efficient and non-parametric classifier. Its classification process is divided into an offline training stage, an online training stage, and a testing stage. Representative samples of different categories are obtained through the first two stages, and these representative samples are called prototypes. However, in the testing stage, the classification of testing samples is completely dependent on the prototype with the maximum similarity, without considering the influence of other prototypes on the classification decision of testing samples. Aiming at the testing stage, this paper proposed a new SOF classifier based on the harmonic mean difference (HMDSOF). In the testing stage of HMDSOF, firstly, each prototype was sorted in descending order according to the similarity between each prototype in the same category and the testing sample. Secondly, multiple local mean vectors of the prototypes after sorting were calculated. Finally, the testing sample was classified into the category with the smallest harmonic mean difference. Based on the above new method, in this paper, the multiscale permutation entropy (MPE) was used to extract fault features, linear discriminant analysis (LDA) was used to reduce the dimension of fault features, and the proposed HMDSOF was further used to classify the features. At the end of this paper, the proposed fault diagnosis method was applied to the diagnosis examples of two groups of different rolling bearings. The results verify the superiority and generalization of the proposed fault diagnosis method.

## 1. Introduction

Rotating machinery has been widely used in various modern industries such as wind turbines, aero engines, water turbines, and gas turbines. As a key component of rotating machinery, rolling bearings play an important role in rotating machinery [1,2,3,4,5]. Due to the complicated structure and harsh operating environment, various faults of rolling bearings (inner ring fault, outer ring fault, ball fault) is inevitable; thus, it is of great significance to study the fault detection methods and diagnostic techniques of rolling bearings [6,7,8,9,10]. In order to find out the fault location, the current frequently used fault diagnosis method is the time-frequency analysis method, such as the variable mode decomposition, local mean mode decomposition, empirical mode decomposition and so on [11,12,13,14].

The fault type is found by the fixed frequency of the intrinsic mode function after time-frequency analysis. However, due to the presence of noise, the collected fault signal may be submerged. In order to enhance the signal, filters are often used, such as minimum entropy deconvolution (MED), maximum correlation kurtosis deconvolution (MCKD), and multipoint optimal minimum entropy deconvolution adjusted (MOMEDA) [15,16,17]. However, the above algorithm is not adaptive, and misdiagnosis may occur. The current intelligent diagnosis method is generally to combine fault extraction technology with the machine learning method, and the first step is to extract fault feature information from the vibration signal [18]. However, since the equipment is usually inevitably operated under friction, vibration, and shock conditions, the vibration signal will show nonlinear and non-stationary characteristics. Since linear analysis methods cannot extract fault features, nonlinear analysis methods are particularly important for fault diagnosis of bearings. In recent years, many nonlinear dynamic methods such as sample entropy (SE), fuzzy entropy (FE), permutation entropy (PE), multiscale sample entropy (MSE), multiscale fuzzy entropy (MFE), multiscale permutation entropy (MPE), and improved methods based on them are used to extract nonlinear fault features [19,20]. For example, Yan et al. [21] extracted bearing fault features with the improved multiscale discrete entropy (MDE) and input them into the extreme learning machine (ELM), obtaining satisfactory fault diagnosis results. Liu et al. [22] used local mean decomposition to denoise the vibration data and then used MSE to extract the fault characteristics from the denoised signal. A lot of research studies have found that MPE has a faster calculation speed and stronger robustness than MSE, MFE, and MDE, and can better extract fault feature information [21,22]. Therefore, this paper uses MPE to extract fault features.

It is well known that after multiscale entropy is used to extract multiscale feature sets, feature reduction is needed to eliminate redundant features and improve computational speed. At present, the commonly used feature dimension reduction methods are principal component analysis (PCA) and linear discriminant analysis (LDA) [23]. For example, Aouabdi et al. [24] used multiscale sample entropy to extract the fault features of gears and then used PCA to reduce the dimensionality of fault features. Chen et al. [25] applied PCA to the feature reduction of high-speed train fault diagnosis. After using empirical mode decomposition to decompose the bearing data into the intrinsic mode function, Su et al. [26] extracted the high-dimensional feature vector set from the intrinsic mode function and then reduced it with LDA. PCA ignores other components while retaining the principal components with larger variance, so critical fault information may be lost during data dimensionality reduction. As a commonly used data dimension reduction method, LDA has a simple principle and a short calculation time. The feature set with dimensionality reduction has more sensitive features and is easier to classify [24,26].

The next and most important step after dimensionality reduction is to input the dimensionality reduction feature set into the classifier. Classification is one of the hot issues in machine learning research. In recent years, various methods of machine learning have been used in the field of fault diagnosis, such as support vector machine (SVM), decision tree (DT), k-nearest neighbor (KNN), extreme learning machine (ELM), etc. [27,28,29,30,31]. The self-organizing fuzzy logic classifier (SOF) has not been used in the field of fault diagnosis since it was proposed in 2018. SOF has the advantages of fast calculation speed, high classification accuracy, and no parameters [32]. The classification process is divided into three stages: the offline training stage, online training stage, and testing stage. In the offline training and online training stage, fuzzy rules of different categories are constructed after various types of qualified prototypes are obtained through self-iterative updating of meta-parameters. However, in the testing stage, the testing samples are classified according to the maximum similarity between the testing samples and the prototypes in each category. This does not take into account the impact of other prototypes in the same category on the classification of testing samples, so classification accuracy may be affected. This paper have improved the testing stage of SOF from the classification decision of SOF. The harmonic mean difference (HMDSOF) proposed in this paper not only considers the influence of other prototypes on testing samples but also assigns different weights to different prototypes. In the experimental part, the influence of the parameter g on the classification result of HMDSOF is analyzed by the bearing fault data of Case Western Reserve University, and the default value of the parameter g is given. Then, by comparing the classification results of HMDSOF with SOF, SVM, DT, KNN, ELM, least squares support vector machine (LSSVM), and kernel extreme learning machine (KELM), the validity and rationality of the proposed HMDSOF are illustrated. Finally, the generalization of HMDSOF is verified by bearing testing data of coal washer.

## 2. Basic Theory

### 2.1. Multiscale Permutation Entropy

MPE can be defined as the set of permutation entropy values of time series at different scales, and its calculation can be described as:

(1) Assuming a one-dimensional time series {x(i),i=1~N} of length *N*. Set the embedded dimension as m and set the delay time as τ, and then conduct phase space reconstruction to obtain the matrix in the following form:

(1) $$\left[\begin{array}{cccc} x(1) & x(1+\tau ) & \cdot \cdot \cdot & x(1+(m-1)\tau )\\ \cdot \cdot \cdot & \cdot \cdot \cdot & \cdot \cdot \cdot & \cdot \cdot \cdot \\ x(j) & x(j+\tau ) & \cdot \cdot \cdot & x(j+(m-1)\tau )\\ \cdot \cdot \cdot & \cdot \cdot \cdot & \cdot \cdot \cdot & \cdot \cdot \cdot \\ x(K) & x(K+\tau ) & \cdot \cdot \cdot & x(K+(m-1)\tau ) \end{array}\right]$$

where K is the number of reconstruction vectors, K=N−(m−1)τ.

To explain Formula (1), let us give an example, assuming that x=(4,8,9,6,5,11,7). When τ=1,m=3, five embedding vectors can be obtained as:

(2) $$\left[\begin{array}{ccc} 4 & 8 & 9\\ 8 & 9 & 6\\ 9 & 6 & 5\\ 6 & 5 & 11\\ 5 & 11 & 7 \end{array}\right].$$

(2) Arrange the reconstruction matrix of each row according to the increasing rule:

(3) $$x(i+(j_{1}-1)\tau )\leq x(i+(j_{2}-1)\tau )\leq \cdots \leq x(i+(j_{m}-1)\tau ).$$

It is important to note that if two equal elements exist in the reconstructed vector, the two elements are arranged in the original order. That is to say, suppose that p and q are any two numbers between 1 and m, if $x(i+(j_{p}-1)\tau )=x(i+(j_{q}-1)\tau )$ and p<q, the following formula can be obtained.

(4) $$x(i+(j_{p}-1)\tau )\leq x(i+(j_{q}-1)\tau )$$

(3) The symbol sequence corresponding to k reconstruction vectors of one-dimensional time series, whose permutation entropy is expressed as:

(5) $$PE_{P}(m)=-\sum_{j=1}^{k}P_{j}\ln P_{j}$$

where $P_{j}$ represents the probability of any time series occurring.

(4) After steps (1), (2), and (3), the permutation entropy of the first scale is calculated. When calculating multiscale permutation entropy, it is necessary to use Formula (6) to conduct multiscale coarse granulation treatment on time series.

(6) $$y_{j}^{\left(s\right)}=\frac{1}{s}\sum_{i=\left(j-1\right)s+1}^{js}x_{i},\;j=1,2,\ldots ,\frac{N}{s}$$

where s=1,2,…, is the scale factor. $y_{j}^{\left(s\right)}$ represents the coarse granulation time series of length $\frac{N}{s}$, it can be seen from Formula (6) that the coarse graining process is achieved by calculating the average value of the time series. $y_{j}^{\left(1\right)}\left(j=1,2,\ldots ,N\right)$ is the original time series.

(5) After coarse granulation of time series, according to steps (1), (2) and (3), permutation entropy of different scales is calculated.

### 2.2. Linear Discriminant Analysis

Theoretically, the extracted multiscale permutation entropy set can be used to identify fault categories. The high-dimensional feature contains a lot of redundant information, so it is necessary to use the dimensionality reduction algorithm to reduce the dimension of the initial, which can not only avoid the dimension disaster, but also improve the performance of fault diagnosis. The role of LDA is to project a high-dimensional matrix into a low-dimensional matrix with minimal intraclass dispersion and maximum interclass dispersion. Assume that the calculated multiscale permutation entropy set is $Y=\left[y_{1},y_{2},\ldots ,y_{n}\right]\in {\mathbb{R}}^{d\times n}$, where n is the total number of samples, d is the dimension, and d=s=32. LDA will supervise the learning of a linear transformation matrix $W\in {\mathbb{R}}^{d\times m}\left(m\ll d\right)$ by itself. After the calculation as below, the high-dimensional data set $y\in {\mathbb{R}}^{d}$ is mapped to the low-dimensional data set $x\in {\mathbb{R}}^{m}$.

(7) $$x=W^{T}y$$

Y is classified as $Y=\left[\varpi_{1},\varpi_{2},\ldots ,\varpi_{C}\right]$, C represents the number of categories. $\varpi_{i}\in {\mathbb{R}}^{d\times n_{i}}$ is the data set of category i, and $n_{i}$ is the number of data samples in the category i.

The optimal projection matrix W should satisfy the following formula:

(8) $$S_{t}^{-1}S_{w}W=W\Lambda$$

where $S_{w}=\sum_{i=1}^{C}\sum_{y\in \pi_{i}}\left(y-\bar{y_{i}}\right){\left(y-\bar{y_{i}}\right)}^{T}$ is defined as an intraclass discrete matrix, $\bar{y_{i}}=\frac{1}{n_{i}}\sum_{y_{j}\in \pi_{i}}y_{j}$. $S_{t}=\sum_{i=1}^{n}\left(y_{i}-\bar{y}\right){\left(y_{i}-\bar{y}\right)}^{T}$ is defined as a discrete matrix of the whole class, $\bar{y}=\frac{1}{n}\sum_{i=1}^{n}y_{i}$, Λ is the eigenmatrix of $S_{t}^{-1}S_{w}$.

### 2.3. Self-Organizing Fuzzy Logic Classifier

SOF is a fuzzy rule classifier without parameters. The algorithm includes three stages: the offline training stage, online training stage, and testing stage. In the first two stages, the fuzzy rules of each category were constructed based on the prototype of each category after the meta-parameters were updated iteratively, and the test samples were classified in the testing stage. The specific process is as follows:

#### 2.3.1. Offline Training Stage

The role of the offline training stage is to find prototypes from different categories and build fuzzy rules that belong to different categories. Suppose there are a total of K samples, (the sample here refers to the low-dimensional feature vector processed by LDA), and the sample set belonging to the category c is ${\left\{x\right\}}_{K^{c}}^{c}=\left\{x_{1}^{c},x_{2}^{c},\ldots ,x_{K^{c}}^{c}\right\}\left({\left\{x\right\}}_{K^{c}}^{c}\subset {\left\{x\right\}}_{K}\right)$, where c=(1,2,…,C). Since the same sample may appear more than once, the unique sample set and the occurrence frequency of each sample in it are expressed as ${\left\{u\right\}}_{U_{K}^{c}}^{c}=\left\{u_{1}^{c},u_{2}^{c},\ldots ,u_{U_{K}^{c}}^{c}\right\}$ and ${\left\{f\right\}}_{U_{K}^{c}}^{c}=\left\{f_{1}^{c},f_{2}^{c},\ldots ,f_{U_{K}^{c}}^{c}\right\}$, respectively. $K^{c}$ is the number of samples of ${\left\{x\right\}}_{K^{c}}^{c}$. It can be concluded that $\sum_{c=1}^{C}K^{c}=K$ and $\sum_{c=1}^{C}U_{K}^{c}=U_{K}$, and $U_{K}$ is the unique sample set for all categories. It should be noted that the selection of the prototypes is carried out under the premise of the same category, and there is no influence between the samples of different categories when selecting the prototypes. The specific process of the offline training stage is as follows:

(1) The multimodal density corresponding to each unique sample is calculated according to Formula (9), where d represents Euclidean distance.

(9) $$D_{K^{c}}^{MM}\left(u_{i}^{c}\right)=f_{i}^{c}\frac{\sum_{l=1}^{K^{c}}\sum_{j=1}^{K^{c}}d^{2}\left(x_{i}^{c},x_{j}^{c}\right)}{2K^{c}\sum_{j=1}^{K^{c}}d^{2}\left(u_{i}^{c},x_{j}^{c}\right)},\;i=1,2,\ldots ,U_{K}^{c}$$

(2) Sort the samples according to the calculated multimode density and mutual distance. The sorted sample set is $\left\{r\right\}=\left\{r_{1},r_{2},\ldots ,r_{U_{K}^{c}}\right\}$, where $r_{1}=\underset{i=1,2,\ldots ,U_{K}^{c}}{\arg\max}\left(D_{K^{c}}^{MM}\left(u_{i}^{c}\right)\right)$, and $r_{2}$ is the sample that has the smallest distance from $r_{1}$, that is, $r_{2}=\underset{i=1,2,\ldots ,U_{K-1}^{c}}{\arg\min\left(d\left(r_{1},u_{i}^{c}\right)\right)}$. $r_{3}$ is a sample with the smallest distance from $r_{2}$, and so on. The multimodal density set of the sorted sample set {r} is represented as $\left\{D_{K^{c}}^{MM}\left(r\right)\right\}$. Then, select the initial prototype according to Formula (10).

(10) $$if\left(D_{K^{c}}^{MM}\left(r_{i}\right)>D_{K^{c}}^{MM}\left(r_{i+1}\right)\right)and\left(D_{K^{c}}^{MM}\left(r_{i}\right)>D_{K^{c}}^{MM}\left(r_{i-1}\right)\right)\;then\left(r_{i}\in {\left\{p\right\}}_{0}\right)$$

where ${\left\{p\right\}}_{0}$ represents a collection of initial prototypes.

(3) In order to increase the number of initial prototypes, the initial prototypes selected by Equation (11) is used as the center to attract nearby samples to form a data cloud.

(11) $$winning\;prototype=\arg\min\left(d\left(x_{i},p\right)\right),p\in {\left\{p\right\}}_{0},x_{i}\in {\left\{x\right\}}_{K^{c}}^{c}$$

It is important to note here that as mentioned above, the sample $x_{i}$ may not be unique, so the data cloud may not consist of only two samples.

(4) Define the set ${\left\{p\right\}}_{0}$ of the initial prototypes obtained by the Formula (10) as ${\left\{\varphi \right\}}_{0}$; that is, define the set of the data cloud center as ${\left\{\varphi \right\}}_{0}$. Recalculate the multimodal density according to Equation (12).

(12) $$D_{K^{c}}^{MM}\left(\varphi_{i}\right)=S_{i}\frac{\sum_{l=1}^{n}\sum_{j=1}^{n}d^{2}\left(\varphi_{l},\varphi_{j}\right)}{2K\sum_{j=1}^{n}d^{2}\left(\varphi_{i},\varphi_{j}\right)}$$

where $\varphi_{i}\in {\left\{\varphi \right\}}_{0}$, $S_{i}$ is the number of samples in the ith data cloud, and n is the number of elements in the set ${\left\{\varphi \right\}}_{0}$.

(5) According to Formula (13), the set ${\left\{\varphi \right\}}_{i}^{neigbor}$ of adjacent centers of each data cloud center is composed.

(13) $$if\left(d^{2}\left(\varphi_{i},\varphi_{j}\right)\leq G_{K^{c}}^{c,L}\right)\;then\left(\varphi_{j}\in {\left\{\varphi \right\}}_{i}^{neigbor}\right)$$

$G_{K^{c}}^{c,L}$ is the average radius of the locally affected area around the data sample corresponding to the level of granularity L, with a default value of L=12. The calculation process is as shown in Equation (14):

(14) $$G_{K^{c}}^{c,L}=\frac{\sum d^{2}\left(x,y\right)}{Q_{K^{c}}^{c,L}}$$

where $x,y\in {\left\{x\right\}}_{K^{c}}^{c},x\neq y,d^{2}\left(x,y\right)\leq G_{K^{c}}^{c,L-1}$, and $G_{K^{c}}^{c,L-1}$ is the average radius of the granularity level L−1. $Q_{K^{c}}^{c,L}$ is the number of times that the distance between any two samples in ${\left\{x\right\}}_{K^{c}}^{c}$ is less than $G_{K^{c}}^{c,L-1}$. $Q_{K^{c}}^{c,1}$ is the number of times that the distance between any two samples in ${\left\{x\right\}}_{K^{c}}^{c}$ is less than the average square distance $\bar{d_{K^{c}}^{c}}$.

(15) $$\bar{d_{K^{c}}^{c}}=\frac{1}{{\left(K^{c}\right)}^{2}}\sum_{l=1}^{K^{c}}\sum_{j=1}^{K^{c}}d^{2}\left(x_{l}^{c},x_{j}^{c}\right)$$

(6) According to Formula (16), select the most representative prototype ${\left\{p\right\}}^{c}$ in the category c from the center of the data cloud.

(16) $$if\left(D_{K^{c}}^{MM}\left(\varphi_{i}\right)>D_{K^{c}}^{MM}\left(\varphi \right)\right)\;then\left(\varphi_{i}\in {\left\{p\right\}}^{c}\right)$$

where $\varphi \in {\left\{\varphi \right\}}_{i}^{neigbor}$.

(7) After determining the representative prototypes of category c, according to Formula (17), AnYa type fuzzy rules belonging to each category are constructed, where $N^{c}$ is the number of prototypes in ${\left\{p\right\}}^{c}$.

(17) $$if\left(x\sim p_{1}^{c}\right)or\left(x\sim p_{2}^{c}\right)or\ldots or\left(x\sim p_{N^{c}}^{c}\right)\;then\left(x\in \left(category\;c\right)\right)$$

where x represents a training sample, and ~ represents similarity.

#### 2.3.2. Online Training Stage

After the offline training stage, it is followed by the input of online training samples to continue training. The purpose of the online training stage is to continue to select prototypes, update the meta-parameters of the classifier, and improve the classification accuracy of the test samples. The online training process is based on the assumption that the samples are stream data that appear one by one. When the online training sample is input, it is assumed that the new sample of the category c is $x_{K^{c}+1}^{c}$, and the sample set after increasing the sample is defined as ${\left\{x\right\}}_{K^{c}+1}^{c}$. In order to improve the computational efficiency, the average radius of the locally affected area will be calculated according to the new formula:

(18) $$G_{K^{c}+1}^{c,L}=\frac{\bar{d_{K^{c}+1}^{c}}}{\bar{d_{K^{c}}^{c}}}G_{K^{c}}^{c,L}.$$

whether the sample $x_{K^{c}+1}^{c}$ is a prototype will be determined according to Formula (19)

(19) $$\begin{array}{l} if\left(\frac{\sum_{l=1}^{K^{c}+1}\sum_{j=1}^{K^{c}+1}d^{2}\left(x_{l}^{c},x_{j}^{c}\right)}{2(K^{c}+1)\sum_{j=1}^{K^{c}+1}d^{2}\left(x_{K^{c}+1}^{c},x_{j}^{c}\right)}>\max\left(\frac{\sum_{l=1}^{mm}\sum_{j=1}^{mm}d^{2}\left(p_{l}^{c},p_{j}^{c}\right)}{2(mm)\sum_{j=1}^{mm}d^{2}\left(p,p_{j}^{c}\right)}\right)\right)or\left(\frac{\sum_{l=1}^{K^{c}+1}\sum_{j=1}^{K^{c}+1}d^{2}\left(x_{l}^{c},x_{j}^{c}\right)}{2(K^{c}+1)\sum_{j=1}^{K^{c}+1}d^{2}\left(x_{K^{c}+1}^{c},x_{j}^{c}\right)}<\min\left(\frac{\sum_{l=1}^{mm}\sum_{j=1}^{mm}d^{2}\left(p_{l}^{c},p_{j}^{c}\right)}{2(mm)\sum_{j=1}^{mm}d^{2}\left(p,p_{j}^{c}\right)}\right)\right)\\ then\left(x_{K^{c}+1}^{c}\in {\left\{p\right\}}^{c}\right) \end{array}$$

where mm is the number of elements in ${\left\{p\right\}}^{c}$, $(p,p_{l}^{c},p_{j}^{c})\in {\left\{p\right\}}^{c}$.

If Formula (19) is not satisfied, we can continue to judge whether the sample $x_{K^{c}+1}^{c}$ is a prototype according to Formula (20).

(20) $$if\left(\min\left(d^{2}\left(x_{K^{c}+1}^{c},p\right)\right)>G_{K^{c}+1}^{c,L}\right)\;then\left(x_{K^{c}+1}^{c}\in {\left\{p\right\}}^{c}\right)$$

If any of Formula (19) or Formula (20) is satisfied, the meta-parameter of the SOF is updated as follows:

(21) $$N^{c}+1\to N^{c},x_{K^{c}+1}^{c}\to p_{N^{c}}^{c},1\to S_{N^{c}}^{c},{\left\{p\right\}}^{c}+p_{N^{c}}^{c}\to {\left\{p\right\}}^{c}.$$

If neither Formulas (19) nor (20) is satisfied, the sample is assigned to the nearest prototype, that is, $p_{n*}^{c}=\arg\min\left(d\left(x_{K^{c}+1}^{c},p\right)\right)$. The corresponding meta-parameters are updated as follows:

(22) $$\frac{S_{n*}^{c}}{S_{n*}^{c}+1}p_{n*}^{c}+\frac{1}{S_{n*}^{c}+1}x_{K^{c}+1}^{c}\to p_{n*}^{c},S_{n*}^{c}+1\to S_{n*}^{c}.$$

After that, Equation (17) will be updated accordingly. The SOF classifier is ready to process the next data sample or enter the testing stage.

#### 2.3.3. Testing Stage

The role of the testing stage is to classify the input testing samples. Assuming that the testing sample set is $\left\{z_{1},z_{2,\ldots }z_{vv}\right\}$, in order to determine the category of a testing sample $z_{ii}$, the classification process of SOF is as follows:

(1) According to Formula (23), calculate the similarity between each prototype selected in the first two stages and the testing sample.
  (23) $$Similarity=e^{-d^{2}\left(z_{ii},p\right)},p\in {\left\{p\right\}}^{c},c=1,2,\ldots ,C,ii=1,2,\ldots ,vv$$
(2) Classify the testing sample into the category of the prototype that has the greatest similarity to the testing sample.
  (24) $$label\left(z_{ii}\right)=\underset{c}{\arg\max}\;Similarity\left(z_{ii},p\right)$$

## 3. Proposed HMDSOF

After the offline training phase and the online training phase, SOF selects a number of representative prototypes from each category of samples, and when selecting prototypes, different categories of samples will not affect each other. However, in the testing stage, the classification of the testing sample is only related to the prototype with the greatest similarity to the testing sample. The effect of other prototypes in the same category on the testing sample classification decisions is not considered, which affects the classification accuracy. In order to improve the classification accuracy, in this paper, we propose a SOF classifier based on harmonic mean difference, which is called HMDSOF. The first two stages of HMDSOF are the same as those of SOF. In the testing stage of HMDSOF, the category of the testing sample is determined and assigned a label corresponding to the category by calculating the harmonic mean difference between the testing sample and each prototype. The content of innovation is mainly two points: (1) The influence of different prototypes in the same category on the classification of the testing sample is considered by calculating multiple local mean vectors in the samples of each category. (2) In order to distinguish the influence of different prototypes in the same category on the testing sample classification decision, the harmonic mean difference constructed by introducing the concept of the harmonic mean is used as the decision of the testing sample classification. In addition, prototypes that differ slightly from test samples have greater weight in their classification decisions. The main process is as follows.

(1) Calculate the similarity between each prototype in each category and the test sample using Equation (22), and arrange the results in descending order.

(2) In each category, the corresponding prototype is sorted according to the result of similarity ranking—that is to say, the prototype with greater similarity to the test sample $z_{ii}$ is sorted in front.

(3) According to Formula (25), calculate the local average vector of the prototype in category c after sorting.

(25) $$a_{i}^{c}=\frac{1}{i}\sum_{h}^{i}p_{h}^{Rc},c=1,2,\ldots ,C$$

where 1≤i≤g, and g is a parameter set before the test stage. In addition, g cannot be bigger than the minimum of the number of prototypes in every category—that is $g\leq \min\left(N^{c}\right)$. It is easy to conclude that g is also the number of local average vectors $a_{i}^{Rc}$, and $a_{1}^{c}=p_{1}^{Rc}$.

(4) Construct the harmonic mean difference by introducing the concept of the harmonic mean value. Suppose there is a sample set $\left\{y_{1},y_{2},\ldots ,y_{g}\right\}$ with g elements, and its harmonic mean value is calculated as shown in Formula (26). The calculation of difference is shown in Formula (27). It can be seen that the value range of the difference is $Difference\left(z_{ii},p\right)\geq 1$. The harmonic mean difference is the sum of the harmonic mean value of the difference between each prototype in the same category and the testing sample. This paper applies the proposed harmonic mean difference to the SOF-based classification decision. The harmonic mean difference is defined as HMD(.), and the calculation process is as shown in Equation (28).

(26) $$HM\left(\left\{y_{1},y_{2},\ldots ,y_{g}\right\}\right)=\frac{g}{\sum_{i=1}^{g}\frac{1}{y_{i}}}$$

(27) $$Difference=e^{d^{2}\left(z_{ii},p\right)},p\in {\left\{p\right\}}^{c},c=1,2,\ldots ,C,ii=1,2,\ldots ,vv$$

(28) $$HMD\left(z_{ii},{\left\{a_{i}^{c}\right\}}_{i=1}^{g}\right)=\frac{g}{\sum_{i=1}^{g}\frac{1}{Difference\left(z_{ii},a_{i}^{c}\right)}}$$

To illustrate the role of harmonic mean differences in assigning different weights to different prototypes in the same category, Equation (29) is given. As can be seen from Equation (29), when setting parameter g, $HMD\left(z_{ii},{\left\{a_{i}^{c}\right\}}_{i=1}^{g}\right)$ is a fixed value. As a result, the prototype that has a smaller difference from the testing sample will be given more weight.

(29) $$\begin{array}{l} \frac{\partial \;HMD\left(z_{ii},{\left\{a_{i}^{c}\right\}}_{i=1}^{g}\right)}{\partial \;Difference\left(z_{ii},a_{i}^{c}\right)}=\frac{\partial \left[\frac{g}{\sum_{i=1}^{g}\frac{1}{Difference\left(z_{ii},a_{i}^{c}\right)}}\right]}{\partial \;Difference\left(z_{ii},a_{i}^{c}\right)}\\ =g\times {\left(1/\left(Difference\left(z_{ii},a_{i}^{c}\right)\times \sum_{i=1}^{g}\frac{1}{Difference\left(z_{ii},a_{i}^{c}\right)}\right)\right)}^{2}\\ =\frac{{\left(HMD\left(z_{ii},{\left\{a_{i}^{c}\right\}}_{i=1}^{g}\right)\right)}^{2}}{g\times {\left(Difference\left(z_{ii},a_{i}^{c}\right)\right)}^{2}}=\frac{1}{g}\times \frac{{\left(HMD\left(z_{ii},{\left\{a_{i}^{c}\right\}}_{i=1}^{g}\right)\right)}^{2}}{{\left(Difference\left(z_{ii},a_{i}^{c}\right)\right)}^{2}} \end{array}$$

(5) Assign the testing sample to the category with the smallest harmonic mean difference. It should be noted that when g=1, Formula (30) can be converted into Formula (24); that is, when g=1, the HMDSOF degenerates into SOF. Equation (31) expresses the relationship between SOF and HMDSOF.

(30) $$label\left(z_{ii}\right)=\underset{c}{\arg\min}HMD\left(z_{ii},{\left\{a_{i}^{c}\right\}}_{i=1}^{g}\right)$$

(31) $$HMD\left(z_{ii},{\left\{a_{i}^{c}\right\}}_{i=1}^{g}\right)=Difference\left(z_{ii},a_{1}^{c}\right)=\frac{1}{Similarity\left(z_{ii},a_{1}^{c}\right)}$$

## 4. Proposed Fault Diagnosis Method

**Figure 1:**
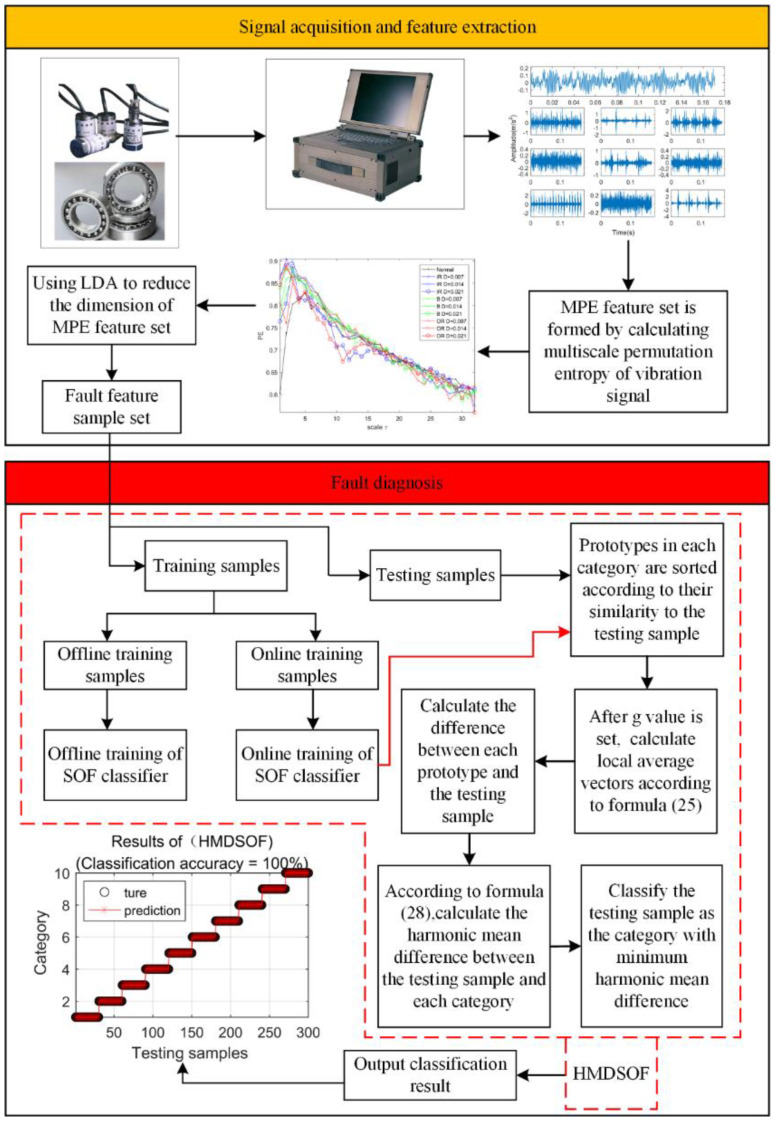
Fault diagnosis flow chart.

The fault diagnosis method proposed in this paper is shown in Figure 1: after the vibration signal is collected, the multiscale permutation entropy set is firstly extracted. The parameters of multiscale permutation entropy selected in this paper are an embedded dimension of m=6 and delay time of τ=1. In order to obtain the signal features as much as possible, the scale factor is set as s=32 [33,34]. It can be seen that such a feature set has many scales and the entropy values are crossed together, which is not conducive to the final classification. Therefore, in this paper, linear discriminant analysis (LDA) is used to conduct dimensionality reduction for the multiscale permutation entropy feature set and the dimension of the feature set after the dimension reduction is nine. Then, the reduced dimensional feature set is randomly divided into online training samples, offline training samples, and testing samples. Finally, the proposed HMDSOF classifier is used for classification. After the training parameters of the HMDSOF are updated in two training stages, the testing samples are classified. For the convenience of description, this fault diagnosis method is named MPE-LDA-HMDSOF.

## 5. Experiments

### 5.1. Experiment 1

**Figure 2:**
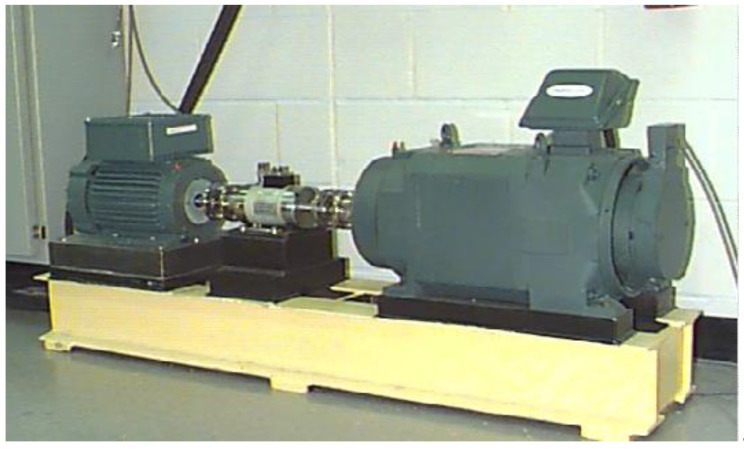
Experimental equipment.

**Figure 3:**
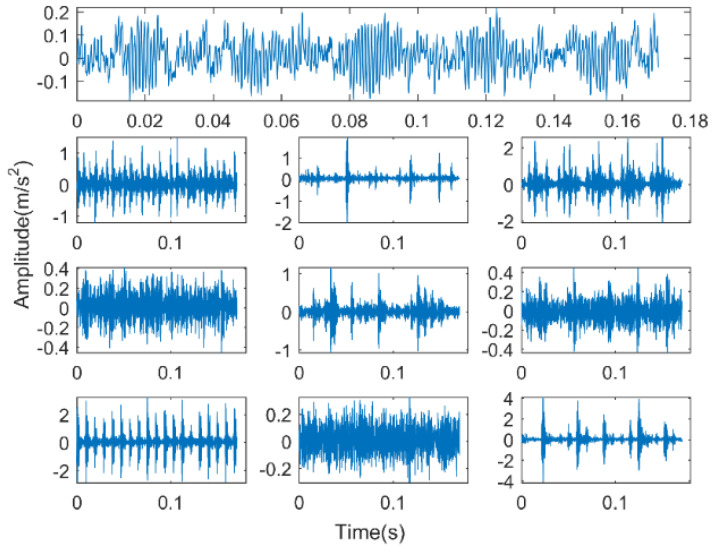
Time-domain diagram of a typical vibration signal of each state of bearing.

**Figure 4:**
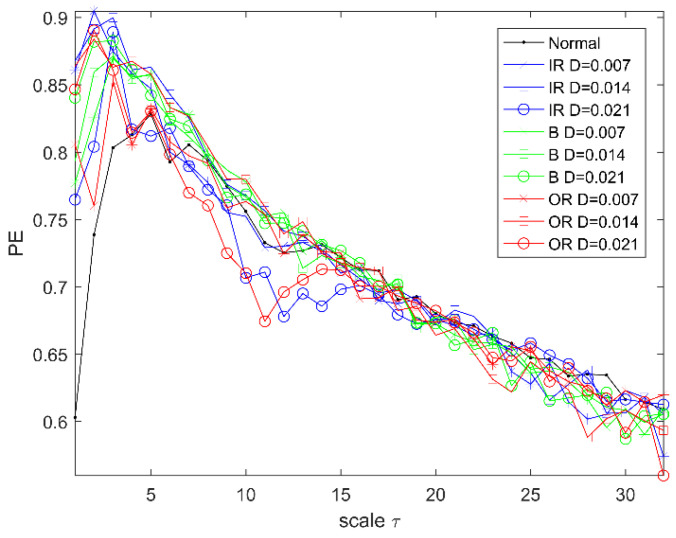
Multiscale permutation entropy.

In Experiment 1, the experimental data of rolling bearings provided by Case Western Reserve University (CWRU) is used to verify the effectiveness of the proposed method. The experimental equipment is shown in Figure 2. It consists mainly of a three-phase induction motor, a torque sensor, and a load motor. The testing bearings are 6205-2RS (SKF, Sweden) deep groove bearings. The vibration acceleration signal of the bearing is obtained from the driving end under the condition of a rotation speed of 1797 r/min and a sampling frequency of 12 kHz. The bearing vibration signals are first classified into four categories, namely ordinary rolling bearings (normal) and rolling bearings with ball failure (B), outer ring failure (OR), and inner ring failure (IR). The faulty bearing is formed on the normal bearing by using electro-discharge machining (EDM), and each fault condition is classified according to the fault size of 0.007, 0.014, and 0.021 inches (1 inch = 25.4 mm), so the bearing vibration signal is finally classified into 10 categories. The first 102,400 points under each category are divided into 50 non-overlapping data samples on average; that is, 2048 sampling points are taken as a sample, and 50 samples can be obtained for each category, for a total of 500 samples. A detailed description of the class label is given in Table 1. The time-domain waveforms of their typical vibration signals are shown in Figure 3. So, a multiscale permutation entropy feature set with the size of 500×32 is obtained. The results of the multiscale permutation entropy corresponding to the vibration signal of Figure 3 are shown in Figure 4. In this paper, 10 samples are randomly selected from each category to form the online training sample set. In the remaining samples, 10 samples are randomly selected in each category to form the offline training sample set, and then the remaining samples constitute the testing sample set. It is known that both the online training set and offline training set have 100 samples, and the test sample set has 300 samples.

The first line in Figure 3 is the time-domain signal corresponding to the normal bearing. The three time-domain waveforms on the left side below correspond to inner ring faults, ball faults, and outer ring faults, and their fault size is 0.007 inches. The three time-domain waveforms on the left side below correspond to inner ring faults, ball faults, and outer ring faults. In addition, their fault size is 0.014 inches. The faulty bearing of the three time-domain waveforms on the right has a fault size of 0.021 inches. In order to facilitate the classification of inner ring type faults of different scales, it is expressed as IRD = 0.007, IRD = 0.014, and IRD = 0.021. Correspondingly, the ball element faults of different sizes are expressed as BD = 0.007, BD = 0.014, and BD = 0.021. Outer ring faults of different sizes are expressed as ORD = 0.007, ORD = 0.014, ORD = 0.021.

Since the experiment in this paper is conducted under the condition of randomly select samples, in order to reduce the impact of contingency, the average value of 10 experiments is taken, and the maximum and minimum values of classification accuracy are given. In addition, the standard deviation of classification accuracy is given to analyze the stability of the classification method. In this paper, three different feature extraction methods (MPE, MPE-PCA, and MPE-LDA) are used to extract fault features and then used for the comparison between SOF and the proposed HMDSOF, and the comparison is listed in Table 2. All the methods are implemented on MATLAB R2016a version and tested on Intel Core CPU i5-6200U @2.30 GHz/4.00 GB RAM and a Win10 computer with a 64-bit operating system.

The contribution rate of each principal component of MPE after PCA treatment is listed in Table 3, and the first eight principal components of the cumulative contribution rate of 90% are selected to form a feature set. Since the minimum number of prototypes of the third category obtained after the end of training in this experiment is 4, the case of g≥5 does not exist. It can be seen that the bigger the value of g is, the longer the classification time will be. When the fault feature extraction method is MPE (numbered 1–4), the average classification time of HMDSOF (g=3) consumes 1.0576 s more than that of SOF. The average classification accuracy of HMDSOF (g=3) is 0.7334% higher than that of SOF, and the standard deviation of the classification accuracy of HMDSOF (g=3) is 0.0005 lower than that of SOF. When the fault feature extraction method is MPE-PCA (numbered 5–8), compared with SOF, the average classification time of HMDSOF (g=3) is 1.0379 s longer, its average classification accuracy is 0.6% higher, and its standard deviation of classification accuracy is 0.0054 lower. When the fault feature extraction method is MPE-LDA (numbered 9–12), compared with SOF, the average classification time of HMDSOF (g=3) is 0.9893 s longer, and the classification accuracy standard deviation is reduced by 0.0022. In addition, the average accuracy of classification was only improved by 0.3667%, but the maximum accuracy of HMDSOF reached 100%, which was satisfactory. When the classification method is HMDSOF and different feature extraction methods are selected (for example, numbered 2, 6, 10, or numbered 3, 7, 11), the comparison of the five indicators shows the advantages of the proposed MPE-LDA-HMDSOF. In conclusion, three different fault extraction methods have shown a better classification effect than SOF after being used as an input of HMDSOF, which proves the effectiveness of the proposed HMDSOF. Under the premise of using the same classification method HMDSOF, the rationality of the proposed fault diagnosis method MPE-LDA-HMDSOF is proved by adopting different classifier inputs. In addition, as the value of g increases, the longer the classification takes, and when g=3, the classification efficiency of the HMDSOF classifier is optimal, so the default value of g is set to 3.

In order to make the proposed HMDSOF more convincing, this paper also compares it with other common classification methods, which are SVM, DT, KNN, ELM, least squares support vector machine (LSSVM), and kernel extreme learning machine (KELM), respectively. The input of each classification method is the features set processed by LDA after calculating multiscale permutation entropy. The training samples of the six classification methods as comparisons are the sum of the online training samples and offline training samples of the HMDSOF, and the test samples used by them are the same as those of HMDSOF. The penalty factor of a standard SVM is 100, and the kernel function is 0.01. The minimum number of father nodes of DT is 5. The nearest neighbor number of KNN is K = 5, and the number of hidden layer nodes of ELM is 100 [21,35]. The Gaussian kernel function of the LSSVM is 0.5. The kernel function of the KELM is RBF, and its regularization parameter is 10,000 [36,37,38,39]. The classification results are shown in Table 4.

It can be seen from Table 4 that the SVM has the lowest classification accuracy, and it can be seen from the standard deviation that the classification effect of this method on different testing samples is very different, and the classification algorithm is very unstable. The standard deviation of the classification accuracy of DT is 1.7525, the algorithm is very unstable, and the minimum classification accuracy is 9% lower than that of HMDSOF. The maximum classification accuracy of KNN is 99%, but the standard deviation of classification accuracy is 1.4915 higher than that of HMDSOF. The input of different samples has a great influence on KNN classification accuracy. The average classification accuracy of ELM is 2.0333% lower than HMDSOF, and the standard deviation of classification accuracy is 0.7316 higher than that of HMDSOF. Compared with SVM, the calculation speed and classification accuracy of LSSVM have been significantly improved. KELM has the fastest calculation speed, but its maximum and minimum classification accuracy are 1% lower than HMDSOF. In addition, from the standard deviation of classification accuracy, the KELM classification stability is not as good as the proposed HMDSOF. In a word, the classification accuracy of HMDSOF is the highest; thus, the classification result is the best.

**Figure 5:**
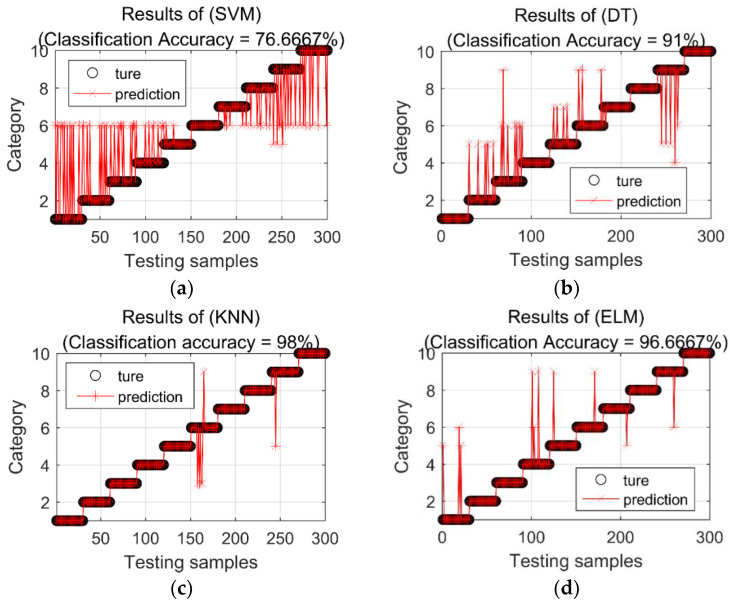

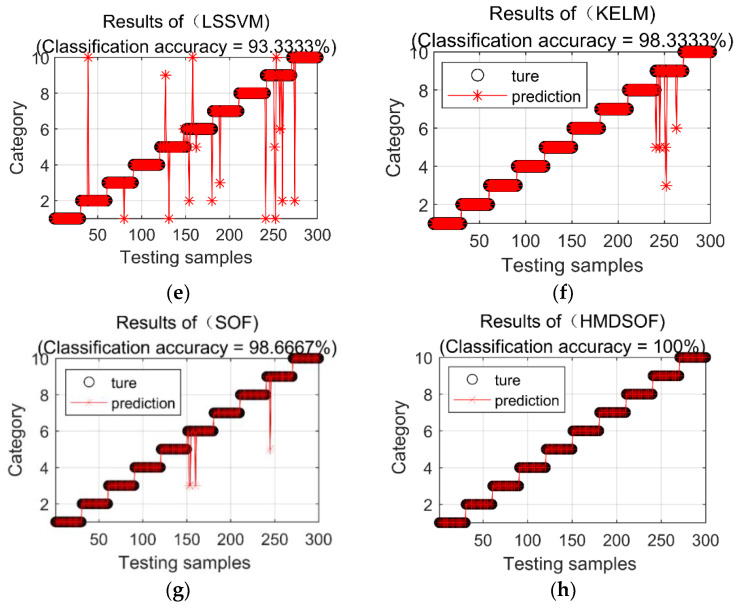
Classification results of each classification method in the fifth experiment. (**a**) Classification results of SVM; (**b**) Classification results of DT; (**c**) Classification results of KNN; (**d**) Classification results of ELM; (**e**) Classification results of LSSVM; (**f**) Classification results of KELM; (**g**) Classification results of SOF; (**h**) Classification results of HMDSOF.

In order to express the classification effects of various classification methods more intuitively, Figure 5 shows the classification results of various classification methods in the fifth experiment. SVM has the lowest classification accuracy. Seventy of the 300 samples do not match the real category. Among the 70 misclassified samples, 67 samples of different categories are classified into category 6, with an overall classification accuracy of 76.6667%. In the classification results of DT, 27 samples are misclassified, and the overall classification accuracy is 91%. In the classification results of KNN, six samples are misclassified, of which four samples in category 6 are classified as category 3, and one sample in category 6 is classified as category 9. In the nine categories, one sample is misclassified as category 5, and the overall classification accuracy of KNN reached 98%. A total of 10 samples in the classification result of ELM are misclassified, and its overall classification accuracy is 91%. In the classification results of SOF, four samples were misclassified, among which three samples in category 6 are classified as category 3, and one sample in category 9 is classified as category 5. The total classification accuracy of SOF is 98.6667%. There are 20 misclassified samples in the classification results of LSSVM, and its classification accuracy is 93.3333%. There are five misclassified samples in the classification results of KELM, and its classification accuracy is 98.3333%. In the classification results of proposed HMDSOF, there are no misclassified samples, and the classification accuracy is 100%.

In addition, in order to evaluate the results of this experiment from different perspectives, F−scores was introduced [40]. Its calculation process is shown in Formulas (32)–(34).

(32) F−scores(j)=2×precision(j)×Recall(j)precision(j)+Recall(j) j=1,2,…,10

(33) $$precision\left(j\right)=\frac{CM_{j,j}}{\sum_{i=1}^{10}CM_{j,i}}$$

**Figure 6:**
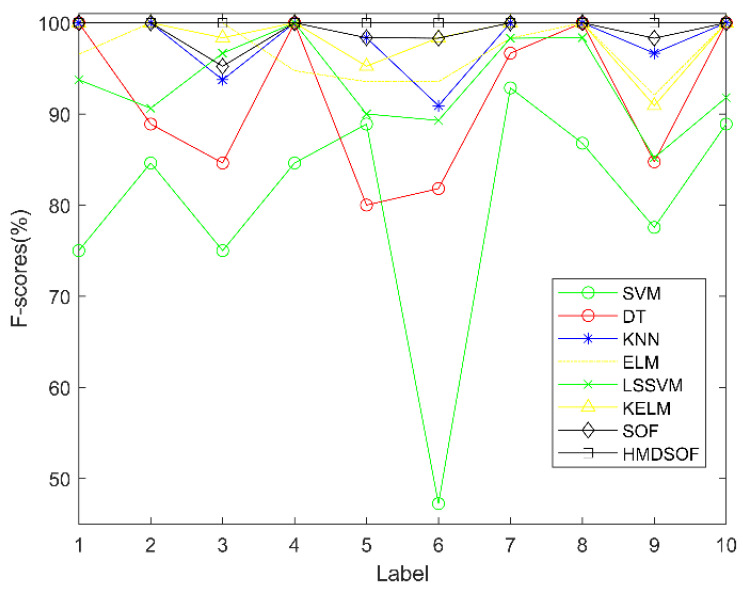
F-scores for each method.

(34) Recall(j)=CMj,j∑i=110CMi,j

where precision(j), Recall(j), and F−scores(j) represent the precision, recall, and F-scores measures of the j-th predicted class; respectively [41]. The F−scores of each category corresponding to the experimental results in Figure 5 is shown in Figure 6.

### 5.2. Experiment 2

The fan-end bearing of CWRU has proven to be a more complex database [35]. In Experiment 2, we use its data to verify the effectiveness of the proposed fault diagnosis method. All the parameters used in Experiment 2 are exactly the same as those in Experiment 1. The classification results of each classification method are shown in Table 5.

### 5.3. Experiment 3

**Figure 7:**
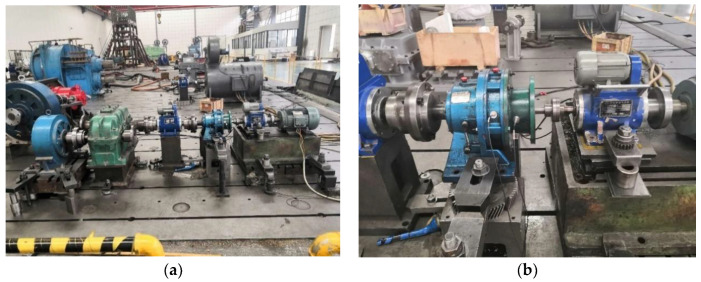
Experimental device and position of measuring points. (**a**) Experimental device; (**b**) position of measuring points.

**Figure 8:**
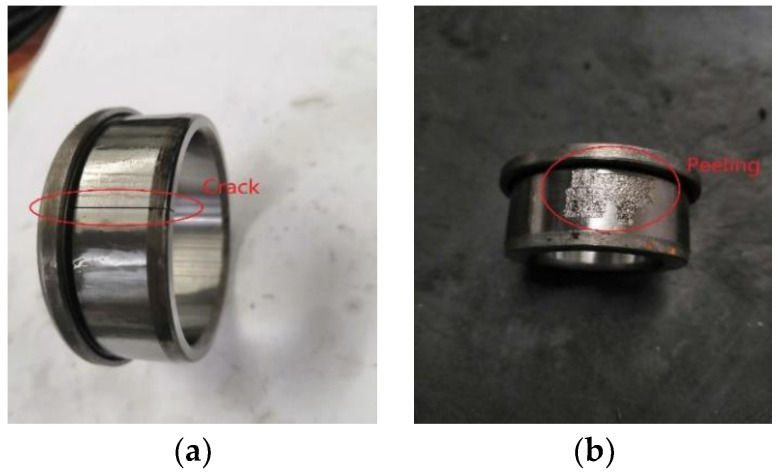
Fault status of two bearings. (**a**) Bearing NJ210 with a crack; (**b**) Bearing NJ405 with a piece of peeling.

**Figure 9:**
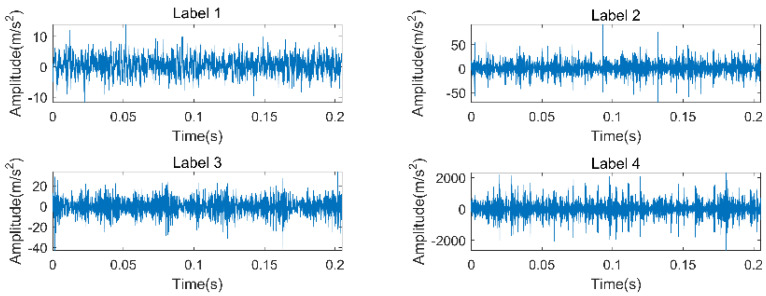
Time-domain diagram corresponding to each state.

This section uses the experimental data of the rolling bearing in the coal washer to verify the generalization of the proposed fault diagnosis method. The experimental device is shown in Figure 7a. The motor speed is 1500 r/min, and the sampling frequency is 10 KHz. There are two acceleration sensors used to measure the bearing signal, and the position of the measuring point is shown in Figure 7b. The two bearing models are NJ210 (NSK, Japan) and NJ405 (NSK, Japan), respectively. NJ210 has two states, normal and crack, and NJ405 also has two states, normal and peeling. Their fault status is shown in Figure 8. In order to distinguish the two bearings, NJ210 is defined as A and NJ405 as B, so the collected signals can be divided into four categories. Their classification is shown in Table 6, and the typical time-domain diagram corresponding to the four states is given in Figure 9.

After calculating the multiscale permutation entropy of the obtained experimental data, LDA is used for dimensionality reduction processing, and the feature set after dimensionality reduction is input into different classification methods for comparison. In this experiment, there are 200 samples for each state, among which 50 samples from each category were randomly selected as the online training samples of HMDSOF and SOF. Then, 50 samples from the remaining 150 samples were randomly selected for offline training, and the remaining 100 samples were used as the testing samples. There are 200 offline training samples, 200 online training samples, and 400 testing samples in HMDSOF and SOF. The training samples in SVM, DT, KNN, and ELM are the sum of the training samples and the offline training samples input to HMDSOF, and their testing samples are the testing samples used by HMDSOF. That is to say, among the four classification algorithms SVM, DT, KNN, and ELM, there are 400 training samples and 400 test samples. The other parameters used in Experiment 3 are the same as those used in Experiment 1, and the comparison results are shown in Table 7.

It can be concluded from Table 7 that among the six classification methods, SVM has the lowest classification accuracy and the worst classification effect. The classification result of KNN is the most unstable, the standard deviation of classification accuracy is the largest, and the classification time is the longest. From the four indicators of classification accuracy, the classification effect of SOF is better than that of SVM, DT, KNN, and ELM. Although the average classification time of HMDSOF is 0.717304 s more than SOF, its maximum classification accuracy is 1.25% higher than SOF, its minimum classification accuracy is 1.5% higher than SOF, its average classification accuracy is 1.375% higher than SOF, and its classification standard deviation is 0.108864 lower than SOF; such results are satisfactory. The standard deviation of the classification accuracy of LSSVM is very close to that of HMDSOF, but the average classification accuracy is 4.1% lower than that of HMDSOF. KELM has the fastest classification speed and the shortest classification time; However, its maximum classification accuracy is 0.5% lower than HMDSOF, and the average classification accuracy is 1.625% lower than HMDSOF.

## 6. Conclusions

In this paper, a new SOF classifier (HMDSOF) based on the harmonic mean difference is proposed. Based on this, a new bearing fault diagnosis method is proposed. The validity and generalization of the proposed fault diagnosis method are verified by the bearing experimental data of Case Western Reserve University and the bearing experimental data of coal washer. The following conclusions can be drawn in this paper.

(1) As the parameter g increases, the classification time of HMDSOF increases. When g=3, the classification effect of HMDSOF is optimal.
(2) Under the premise of the same input, the proposed classification effect of HMDSOF is always higher than that of SOF, and the classification effect is better. By comparing with SVM, DT, KNN, ELM, LSSVM, and KELM, the proposed HMDSOF has higher classification accuracy and can be better used for bearing fault diagnosis.
(3) By changing the input of the classifier, it is proved that the proposed bearing fault diagnosis method MPE-LDA-HMDSOF has better classification performance, and the classification accuracy reaches 100%.

## Figures and Tables

**Table 1:** Labels for various states. B: Ball failure, IR: inner ring failure, OR: outer ring failure.

Stage	Fault Size (Inches)	Label	Stage	Fault Size (Inches)	Label
Normal	0	1	B	0.014	6
IR	0.007	2	OR	0.014	7
B	0.007	3	IR	0.021	8
OR	0.007	4	B	0.021	9
IR	0.014	5	OR	0.021	10

**Table 2:** Classification results. HMDSOF: harmonic mean difference, LDA: linear discriminant analysis, MPE: multiscale permutation entropy, PCA: principal component analysis, SOF: self-organizing fuzzy.

Serial Number	Methods	Classification Accuracy (%)				Time/s
		Maximum	Minimum	Average	Std	
1	MPE-SOF	98.3333	94.3333	96.7333	0.0139	0.4243
2	MPE-HMDSOF (g=2)	98.6667	94.3333	97.2333	0.0139	1.3394
3	MPE-HMDSOF (g=3)	98.6667	94.6667	97.4667	0.0134	1.4819
4	MPE-HMDSOF (g=4)	98.6667	94.6667	97.4667	0.0134	1.6589
5	MPE-PCA-SOF	98.6667	95	97.4	0.0128	0.5149
6	MPE-PCA-HMDSOF (g=2)	98.6667	95	97.8333	0.0114	1.3203
7	MPE-PCA-HMDSOF (g=3)	99	97	98	0.0074	1.5528
8	MPE-PCA-HMDSOF (g=4)	99	97	98	0.0074	1.7343
9	MPE-LDA-SOF	99.3333	96.6667	98.6333	0.0074	0.4532
10	MPE-LDA-HMDSOF (g=2)	99.3333	97.3333	98.8333	0.0059	1.2799
11	MPE-LDA-HMDSOF (g=3)	100	98.3333	99	0.0052	1.4425
12	MPE-LDA-HMDSOF (g=4)	100	98.3333	99	0.0052	1.5943

**Table 3:** Results of PCA dimension reduction.

Principal Component	Eigenvalue (×10^−4^)	Rate of Contribution %	Cumulative Contribution Rate %	Principal Component	Eigenvalue (×10^−4^)	Rate of Contribution %	Cumulative Contribution Rate %
1	105	64.7027	64.7027	17	0.7270	0.4480	95.1333
2	23	14.1730	78.8757	18	0.7093	0.4371	95.5704
3	7.3132	4.5065	83.3822	19	0.6739	0.4153	95.9857
4	4.3134	2.6580	86.0402	20	0.6557	0.4041	96.3898
5	2.3684	1.4594	87.4996	21	0.6444	0.3971	96.7869
6	1.6289	1.0038	88.5034	22	0.6092	0.3754	97.1623
7	1.3030	0.8029	89.3063	23	0.5796	0.3572	97.5195
8	1.2143	0.7483	90.0546	24	0.5147	0.3172	97.8367
9	1.1439	0.7049	90.7595	25	0.4998	0.308	98.1447
10	1.0941	0.6742	91.4337	26	0.4887	0.3011	98.4458
11	0.9919	0.6112	92.0449	27	0.4816	0.2968	98.7426
12	0.9173	0.5653	92.6102	28	0.4705	0.2899	99.0325
13	0.8805	0.5426	93.1528	29	0.4342	0.2676	99.3001
14	0.8576	0.5285	93.6813	30	0.4059	0.2501	99.5502
15	0.8398	0.5175	94.1988	31	0.3827	0.2358	99.786
16	0.7895	0.4865	94.6853	32	0.3476	0.2142	100.0002

**Table 4:** Classification results of various methods. DT: decision tree, ELM: extreme learning machine, KNN: k-nearest neighbor, LSSVM: least squares support vector machine, KELM: kernel extreme learning machine, SVM: support vector machine.

Classification Method	Classification Accuracy (%)				Time/s
	Maximum	Minimum	Average	std	
SVM	79.3333	73.3333	76.5667	1.8682	0.8304
DT	95.3333	89.3333	92.5333	1.7525	1.556
KNN	99	94	98.5333	1.4967	0.7318
ELM	97.6667	95	96.9667	0.7371	0.1348
LSSVM	94.6667	89.6667	92.6	1.4126	0.1149
KELM	99	97.3333	98.3	0.4583	0.0344
HMDSOF (g=3)	100	98.3333	99	0.0052	1.4425

**Table 5:** Classification results of various methods.

Input of Classifier	Classification Methods	Classification Accuracy (%)				Time/s
		Maximum	Minimum	Average	Std	
MPE-LDA	SVM	84	78.3333	81.9667	1.456611	0.761977
	DT	92.3333	85	89.4333	2.049457	1.845077
	KNN	94	91	92.3667	0.874959	0.765174
	ELM	91	87.3333	89.2	1.146975	0.137345
	SOF	94	90.3333	92.25	1.056215	0.504949
	LSSVM	87.3333	80	84.2	2.459441	0.121701
	KELM	93.6667	91.3333	92.7333	0.711821	0.025278
	HMDSOF (g=3)	97.3333	92.6667	94.4	1.27191	1.791814

**Table 6:** Category labels for various states.

Label	Corresponding State
1	A is normal, B is normal
2	A has a crack, B is normal
3	A is normal, B has a piece of peeling failure
4	A has a crack, B has a piece of peeling failure

**Table 7:** Classification results of various methods.

Input of Classifier	Classification Methods	Classification Accuracy (%)				Time/s
		Maximum	Minimum	Average	Std	
MPE-LDA	SVM	95.75	91.25	93.85	1.146734	0.601842
	DT	95.75	93.75	94.775	0.719809	1.643364
	KNN	97.75	91.5	96.05	1.627114	2.029434
	ELM	96.75	94.25	95.9	0.845577	0.17406
	SOF	98	96.25	97.05	0.556776	0.426295
	LSSVM	95	93.5	94.325	0.447911	0.095361
	KELM	98.75	95.75	96.8	1.15	0.029672
	HMDSOF (g=3)	99.25	97.75	98.425	0.447912	1.143599
